# The Role of Delayed Interval Debulking Surgery (DIDS) in the Surgical Treatment of Advanced Epithelial Ovarian Cancer: A Retrospective Cohort from an ESGO-Certified Center †

**DOI:** 10.3390/medsci13040217

**Published:** 2025-10-02

**Authors:** Dimitrios Zouzoulas, Iliana Sofianou, Panagiotis Tzitzis, Vasilis Theodoulidis, Kimon Chatzistamatiou, Eleni Timotheadou, Grigoris Grimbizis, Dimitrios Tsolakidis

**Affiliations:** 1 1st Department of Obstetrics & Gynecology, Aristotle University of Thessaloniki, “Papageorgiou” Hospital, 56403 Thessaloniki, Greece; 2 Department of Oncology, Aristotle University of Thessaloniki, “Papageorgiou” Hospital, 56403 Thessaloniki, Greece

**Keywords:** ovarian cancer, neoadjuvant chemotherapy, delayed interval debulking surgery

## Abstract

**Background/Objectives**: Patients with advanced ovarian cancer with a high tumor burden typically undergo neoadjuvant chemotherapy (NACT) followed by interval debulking surgery. The optimal number of NACT cycles remains undefined: although three to four cycles are considered gold-standard, in real-world practice, five or more cycles are frequently administrated. This study aims to evaluate the impact of delayed interval debulking surgery (DIDS) after ≥5 cycles of NACT on the survival rates. **Methods**: We conducted a retrospective analysis of women with advanced ovarian cancer that underwent surgery in the 1st Department of Obstetrics–Gynecology Clinic from 2012 to 2022. Patient characteristics, oncological, and follow-up information were collected. **Results**: A total of 125 patients met the inclusion criteria and were divided into two groups: Group A (77 patients) received 3–4 of NACT cycles, and Group B (48 patients) ≥5 cycles. No statistically significant difference was observed between the groups concerning age, BMI, comorbidities, Aletti score, FIGO stage, pre-operative CA-125 values, surgery duration, rate of postoperative complications, hospital stay, ICU admittance, and complete gross resection (RD = 0). However, patients undergoing DIDS experienced significantly greater intraoperative blood loss. Progression-free survival did not differ between groups (IDS: 17 vs. DIDS: 18 months, *p* = 0.561), whereas overall survival was significantly lower in the DIDS group (IDS: 52 vs. DIDS: 36 months, *p* = 0.00873). This statistical significance persisted after controlling for residual disease, but was lost after adjusting for FIGO stage. **Conclusions**: DIDS may be considered for advanced ovarian cancer patients with a high tumor burden, when complete gross resection (RD = 0) cannot be achieved during IDS. Further prospective randomized trials are necessary to evaluate its oncological safety and morbidity.

## 1. Introduction

Ovarian cancer is the third most common gynecological malignancy and the leading cause of death among women with gynecological cancers [1]. It is usually diagnosed at an advanced stage, resulting in poorer survival outcomes [2]. The standard treatment for advanced ovarian cancer consists of debulking surgery combined with systematic chemotherapy. Primary debulking surgery (PDS) followed by adjuvant chemotherapy is the cornerstone of treatment [3]. However, frail patients, patients with high tumor burden or extra-abdominal metastases may benefit from neoadjuvant chemotherapy (NACT) followed by interval debulking surgery (IDS). In recent years, three randomized non-inferiority trials [4,5,6] have demonstrated that NACT is a valid alternative to PDS. Similarly, the preliminary results from the TRUST trial [7] showed that the primary endpoint of overall survival (OS) was not met, with numerically longer OS in the PDS group compared to NACT (PDS: 54.3 months vs. IDS: 48.3 months) in non-frail ovarian cancer patients. The goal for advanced ovarian cancer treatment is to achieve zero residual disease (RD) after surgery, irrespectively of the timing and type of systematic therapy, with acceptable postoperative complications and minimal patient quality of life decrease. So, the standard treatment for advanced ovarian cancer is upfront surgery followed by chemotherapy for eligible patients; however some may benefit from a change in the timing of surgery, placed in-between the cycles of chemotherapy. Recently, studies have focused on the patients that cannot achieve zero residual disease after three to four cycles of NACT and need further chemotherapy administration, followed by delayed interval debulking surgery (DIDS).

The optimal number of NACT cycles has not yet been established. Most studies in the literature recommend three to four cycles. However, in real-world clinical practice, some patients receive five or more cycles before surgery. The role of DIDS, defined as debulking surgery after five or more cycles of NACT, is yet to be fully investigated and is not presented in current guidelines for advanced ovarian cancer surgical management. Nevertheless, patients with high tumor load, especially in the upper abdomen, could benefit from prolonged NACT followed by DIDS. An ongoing multicenter, randomized phase III trial (CHRONO) is currently investigating whether three or six cycles of NACT before cytoreduction impact progression-free survival (PFS) [8]. In most cases, extended NACT is administrated due to poor patient performance status or most often due to the anticipated inability to achieve complete gross resection (RD = 0) at surgery, which remains the most significant independent prognostic factor [9]. Furthermore, 15–20% of all advanced ovarian cancer patients are poor responders to systematic chemotherapy [10], making it essential to identify this subgroup. Several tools have been proposed for assessing tumor resectability, including image evaluation by Response Evaluation Criteria in Solid Tumors (RECIST) [11], diagnostic laparoscopy [12], and KELIM score [13], all of which showed promising results. For patients with minor radiological or clinical response to chemotherapy, DIDS is proposed as an alternative. The rationale is to allow for maximal tumor shrinkage and improved likelihood of complete gross resection (RD = 0), while reducing surgical complexity and perioperative morbidity. Nonetheless, high-quality prospective data are lacking, and the optimal number of NACT cycles before cytoreduction remains uncertain.

The primary objective of our study was to evaluate the effect of DIDS on progression-free survival, overall survival, and perioperative morbidity in patients with advanced ovarian cancer, particularly among women deemed unfavorable candidates for IDS after standard NACT.

## 2. Materials and Methods

### 2.1. Study Characteristics

We retrospectively reviewed the medical records from all women who underwent surgery for advanced ovarian cancer at the Gynecological–Oncology Unit of the 1st Department of Obstetrics and Gynecology, Aristotle University of Thessaloniki (AUTh), “Papageorgiou” General Hospital, between 1 January 2012 and 31 December 2022. From this cohort, we selected women who had received NACT followed by cytoreductive surgery. A total of 324 patients were identified during this period and were initially included in the study.

### 2.2. Patients

Inclusion criteria:
  - Age at diagnosis ≥ 18 years.
  - Histologically confirmed epithelial ovarian carcinoma.
  - Patients ultimately underwent surgery at out Gynecological–Oncology Unit.
Exclusion criteria:
  - Primary debulking surgery.
  - Recurrent ovarian cancer.
  - Incomplete or missing registry data.

**Figure 1:**
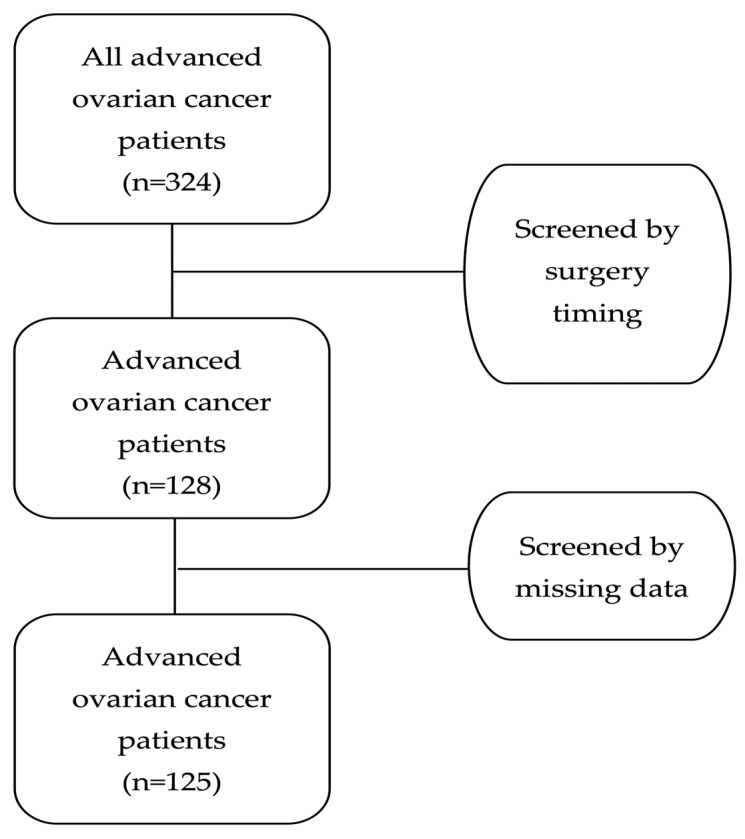
Patient selection flowchart.

After applying these criteria, 196 of the 324 patients were excluded due to having undergone upfront surgery without NACT or because their diagnosis represented a disease relapse. In addition, three women were excluded due to missing follow-up data. Therefore, the final study cohort consisted of 125 patients with newly diagnosed advanced epithelial ovarian cancer. Figure 1 shows the patient selection flowchart of the study. The dataset contained no duplicate records and no clinically relevant missing data.

### 2.3. Ethics

This study was approved by the Institutional Review Board of “PAPAGEORGIOU” General Hospital before its commencement (Νο. 2056, date of approval: 6 September 2024). Moreover, informed consent was waived by the Institutional Review Board, as this study involved a retrospective review of medical records with no direct contact with patients, and all data were already routinely collected and available at the time of the study.

### 2.4. Data Collection

Data collection extended over a period of one month. The Gynecological–Oncology Unit maintains an electronic registry containing comprehensive patient medical records. To ensure consistency, data abstraction was performed using a standardized extraction sheet (Excel format). The following variables were retrieved:

- Patient identification number;
- Age at diagnosis;
- Body Mass Index (BMI);
- Charlson Comorbidity Index (CCI);
- Number of NACT cycles;
- Histological types;
- Aletti score;
- CA-125 level;
- FIGO classification (2014 International Federation of Gynecology and Obstetrics staging system);
- Intraoperative blood loss;
- Duration of surgery;
- Intensive Care Unit (ICU) admission;
- Clavien–Dindo classification for postoperative complications;
- Hospital stay length;
- Residual disease (RD) after cytoreduction;
- Timeline data:
  ○ Date of surgery;
  ○ Date of recurrence;
  ○ Date of last follow-up or death.

Patients were divided into two groups according to the number of NACT cycles received prior to debulking surgery. Group A consisted of women that underwent three or four (3–4) cycles, while patients in Group B were offered five or more (≥5) cycles of NACT. Patients in Group A underwent IDS and patients in Group B were treated with DIDS.

### 2.5. Statistical Analysis

All analyses were performed using R software, version 2025.09.0+387. For categorical variables, descriptive statistics were expressed as absolute frequencies and percentages. For continuous variables, central tendency (mean, median) and dispersion (range, standard deviation) were reported. Progression-free survival (PFS) and overall survival (OS) were estimated using the Kaplan–Meier method, and group differences were assessed by the log-rank test. Univariate and multivariate analyses were conducted using the Cox proportional hazards regression models. PFS was identified as the time period from the date of surgery to the date of first documented recurrence or disease progression. OS was identified as the time period from surgery to death from any cause or last follow-up. A two-sided *p*-value < 0.05 was set for statistical significance.

## 3. Results

This retrospective cohort initially included 324 women with advanced epithelial ovarian cancer who underwent cytoreductive surgery during the specified period at the Gynecological–Oncology Unit of the 1st Department of Obstetrics and Gynecology, Aristotle University of Thessaloniki (AUTh), ‘Papageorgiou’ General Hospital. After applying inclusion and exclusion criteria, 125 patients were eligible for analysis. Patients were offered NACT with the classic chemotherapy regiments of Carboplatin plus Paclitaxel every 3 weeks. Maintenance therapy was administrated only after cytoreductive surgery, based on the individual characteristics of the disease and performance status of each patient.

**Figure 2:**
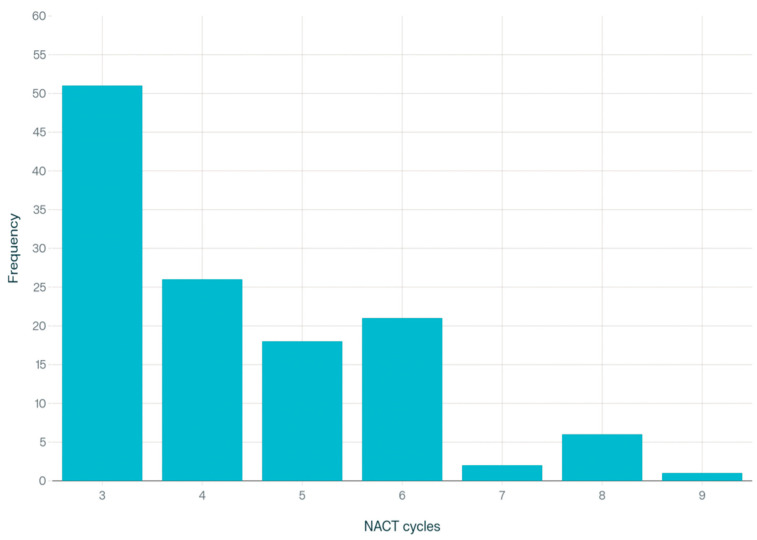
Number of NACT cycles.

Baseline characteristics are summarized in Table 1. The mean age of the patients was 60.8 years old and the mean Body Mass Index (BMI) was 28.1 kg/m^2^, indicating an overweight population. Concerning the performance status of the patients that underwent IDS, the majority of them (85.6%) had mild-to-moderate comorbidities based on the Charlson Comorbidity Index (CCI). Moreover, over two-thirds of them had high-grade serous ovarian cancer. Most of the patients (87.2%) had a low-to-moderate Aletti score at the time of debulking surgery. In addition, median intraoperative blood loss and median surgery duration were 300 mL (IQR: 200–500) and 230 min (IQR: 180–300), respectively. The median preoperative CA-125 value was in normal ranges at 32 IU/mL (IQR: 14.7–117), which is in accordance with the literature, where in most cases CA-125 values decrease after each cycle of NACT. Postoperative complications assessed by using the Clavien–Dindo classification system has a median of 24.2 (IQR: 12.2–32). Only 28 patients (22.4%) required Intensive Care Unit (ICU) admission and the median length of hospital stay was 8 days (IQR: 7–9). Complete gross resection (RD = 0) after debulking surgery was achieved in 72.8%, while only 8% had an incomplete cytoreduction (residual disease ≥1). Nearly two-thirds of the patients (61.6%) received 3–4 cycles of NACT followed by IDS, and 38.4% received ≥5 cycles of NACT followed by DIDS. The exact distribution of NACT cycles is shown in Figure 2.

Based on the number of NACT cycles, the cohort was divided into two groups: Group A with 77 patients (61.6%) who received three-to-four cycles followed by IDS, and Group B with 48 patients (38.4%) who were offered five or more cycles followed by DIDS. The two groups were similar in terms of baseline characteristics and surgical outcomes. No statistically significant differences were found in age, BMI, comorbidities, FIGO stage, Aletti score, histology, preoperative CA-125 values, surgery duration, postoperative complications, ICU admission, length of hospital stay, or rate of residual disease. Although, patients with FIGO stage IV disease were more likely to undergo DIDS, and ICU admission was more frequent, these differences were not statistically significant. Furthermore, the surgical complexity (Aletti score: median 4 in both groups) and the complete gross resection (RD = 0) rate (IDS: 70.1% vs. DIDS: 77.1%) were also comparable, suggesting homogeneity between groups. On the other hand, the only significant difference was found in intraoperative blood loss (IDS: 300 mL vs. DIDS: 450 mL), indicating higher hemorrhage risk in the DIDS group. The aforementioned data are presented in detail in Table 2.

**Figure 3:**
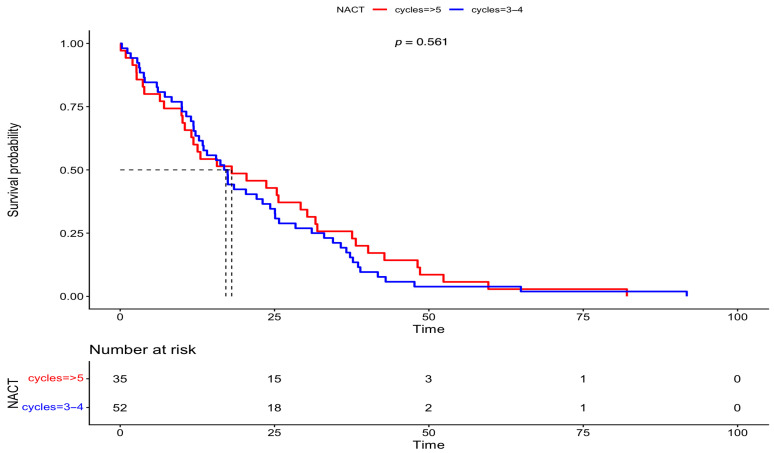
Progression-free survival comparison between IDS vs. DIDS (Kaplan–Meier curve).

**Figure 4:**
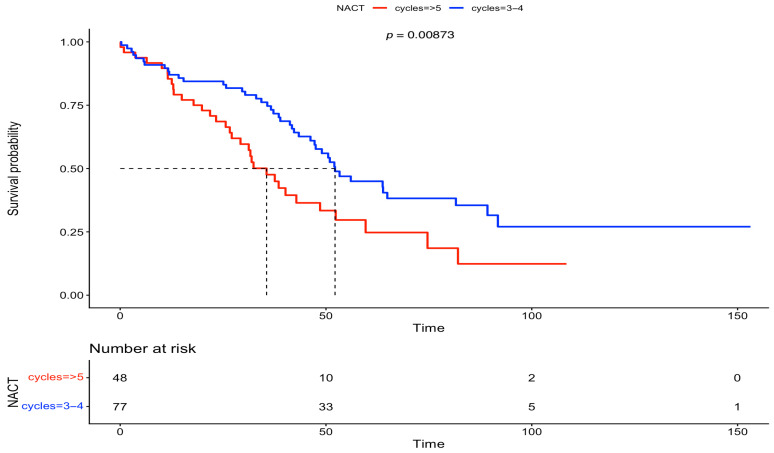
Overall survival comparison between IDS vs. DIDS (Kaplan–Meier curve).

The patients of this cohort were part of a frequent monitoring program, with a mean follow-up period of 43.7 months. Specifically, patients in Group A that underwent IDS had a median follow-up of 45.7 months, while patients in Group B that underwent DIDS had a shorter follow-up of 31.5 months. Survival outcomes were estimated using Kaplan–Meier survival analysis. The median PFS in Group A (IDS) and Group B (DIDS) was 17 and 18 months, respectively. Meanwhile, the median OS in Group A (IDS) and Group B (DIDS) was also 52 and 36 months, respectively. Differences in survival between the two groups were assessed by log-rank tests; no statistical significance was discovered in PFS (*p* = 0.561); however, a statistically significant difference was observed in OS (*p* = 0.00873). These results are presented in Figure 3 and Figure 4.

**Figure 5:**
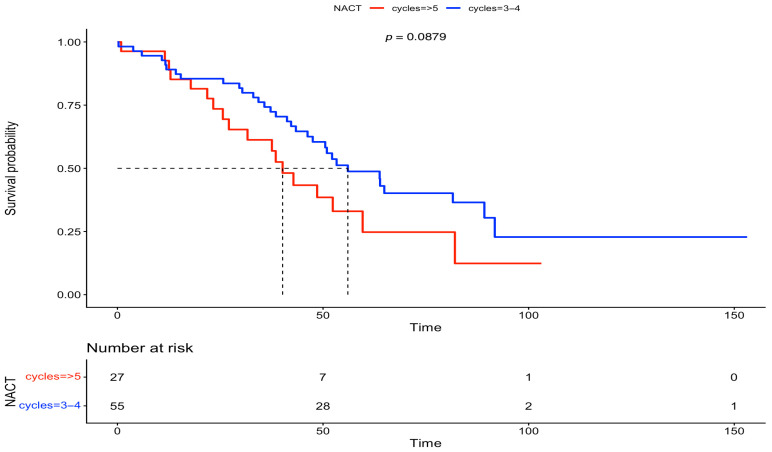
Overall survival comparison between IDS vs. DIDS, stratified by FIGO stage III (Kaplan–Meier curve).

**Figure 6:**
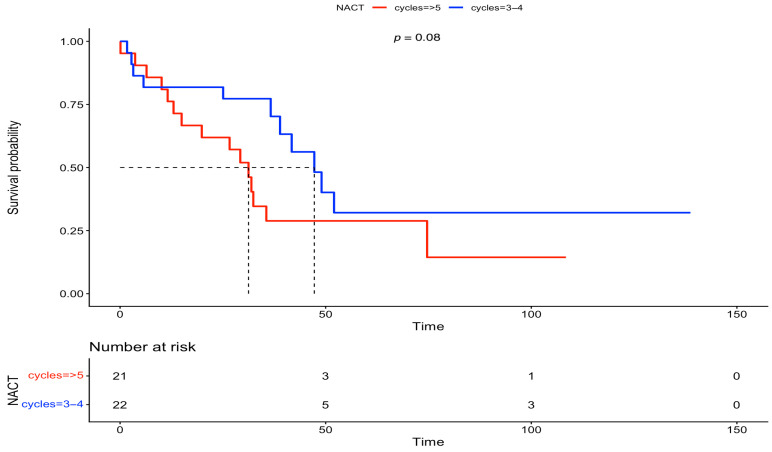
Overall survival comparison between IDS vs. DIDS, stratified by FIGO stage IV (Kaplan–Meier curve).

**Figure 7:**
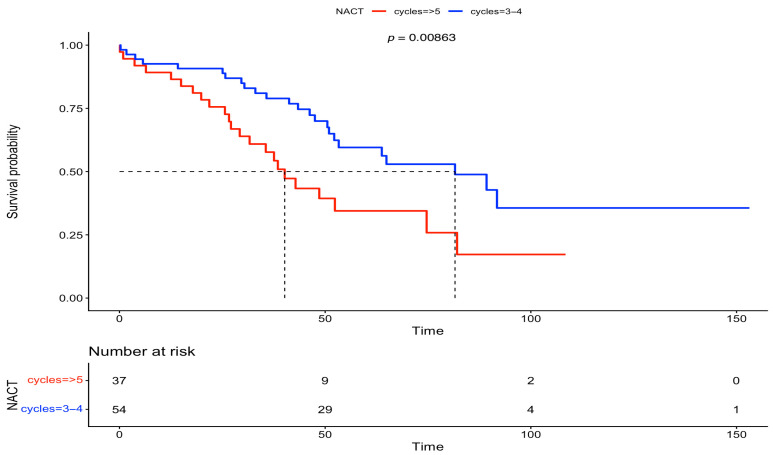
Overall survival between IDS vs. DIDS in patients with no residual disease (Kaplan–Meier curve).

**Figure 8:**
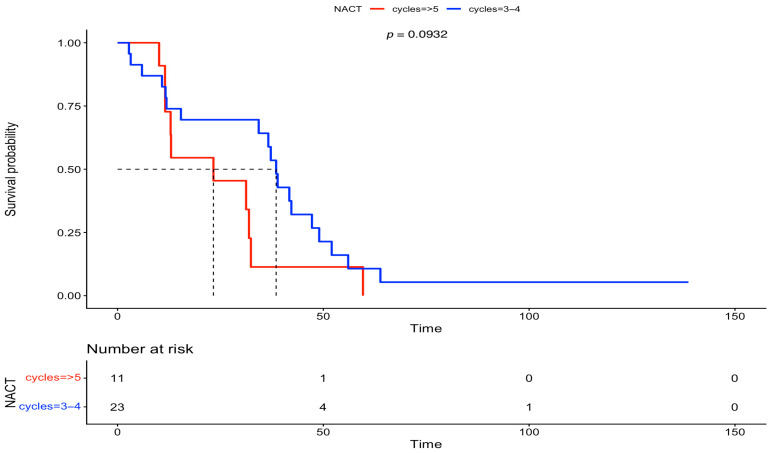
Overall survival between IDS vs. DIDS in patients with any residual disease (Kaplan–Meier curve).

Furthermore, we analyzed the OS between the two groups, based on FIGO stage and residual disease. There was no statistically significant difference in OS at FIGO stage III (IDS: 56 months vs. DIDS: 40 months, *p* = 0.0879) and at FIGO stage IV (IDS: 47 months vs. DIDS: 31 months, *p* = 0.08). On the other hand, after analyzing the OS based on residual disease, a statistically significant difference was observed in both subgroups with complete gross resection (RD = 0) (IDS: 82 months vs. DIDS: 40 months, *p* = 0.00863), but statistical significance was lost for any residual disease (RD > 0) (IDS: 38 months vs. DIDS: 23 months, *p* = 0.0932). The above-mentioned survival data are outlined in Figure 5, Figure 6, Figure 7 and Figure 8.

Last but not least, univariate and multivariate analyses were conducted using Cox regression to identify potential independent predictors of survival. In the univariate analyses for the risk of recurrence, statistical significance was observed in FIGO stage (HR 1.69, 95% CI: 1.07–2.68, *p* = 0.0258), Aletti score (HR 1.14, 95% CI: 1.02–1.27, *p* = 0.0167), surgery duration (HR 1.004, 95% CI: 1.001–1.01, *p* < 0.01), postoperative complications (HR 1.02, 95% CI: 1.007–1.04, *p* < 0.01), and residual disease (HR 3.51, 95% CI: 1.68–7.35, *p* < 0.01). On the other hand, in the univariate analyses for the risk of death, statistical significance was present in NACT cycles (HR 1.83, 95% CI: 1.16–2.9, *p* < 0.01), CA-125 values (HR 1, 95% CI: 1–1.001, *p* = 0.018), intraoperative blood loss (HR 1.001, 95% CI: 1–1.001, *p* < 0.01), and residual disease (HR 0.23, 95% CI: 0.11–0.47, *p* < 0.01). In the multivariate analyses, after adjusting for all possible confounders, postoperative complication was independently related to the risk of recurrence (HR 1.03, 95% CI: 1.01–1.05, *p* < 0.01) and death (HR 1.02, 95% CI: 1.002–1.04, *p* < 0.01). Similarly, residual disease was independently associated with increased risk of relapse (HR 2.84, 95% CI: 1.33–6.06, *p* < 0.01) and mortality (HR 0.18, 95% CI: 0.08–0.38, *p* < 0.01). Specifically, patients with residual disease ≥1 cm, had almost three times higher risk of recurrence, while patients with no residual disease (0 cm) had an 82% lower risk of death compared to those with residual disease. Furthermore, the total number of NACT cycles was identified as an independent factor only for mortality (HR 1.84, 95% CI: 1.15–2.96, *p* = 0.0116), showing the patients that underwent DIDS had an 84% higher risk of death compared to those that underwent IDS. The results are shown in Table 3 and Table 4.

## 4. Discussion

The present study was conducted in a tertiary university clinic, which is an ESGO-certified center for advanced ovarian cancer surgery. Such centers combine high surgical expertise, multidisciplinary care, access to innovative therapies, and rigorous quality assurance, thereby enhancing the reliability, generalizability, and clinical impact of our results. Treatment at high-volume, accredited centers is associated with better overall survival and quality of life compared to non-specialized hospitals [14]. We investigated the role of DIDS following NACT in patients with advanced epithelial ovarian cancer. In patients unfit for primary cytoreduction, the gold-standard remains 3–4 cycles of NACT followed by cytoreductive surgery. However, real-world data showed that many patients undergo ≥5 cycles before debulking surgery, often due to poor initial response to chemotherapy, comorbidities, or frailty. In our study, the decision for additional cycles of NACT followed by DIDS was based mainly based on tumor resectability, which was assessed either by computerized tomography (CT) or diagnostic laparoscopy, or the decrease of CA-125 (KELIM score). Other factors affecting the decision for DIDS were patient frailty and the lack of available ICU beds, especially during the Coronavirus Disease 2019 (COVID-19) pandemic. The primary outcome was to assess the impact of DIDS on PFS and OS retrospectively in our center population.

The primary finding was a statistically significant reduction in OS among patients undergoing five or more cycles of NACT followed by DIDS compared to those treated with three to four cycles of NACT followed by IDS, whereas PFS did not significantly differ between groups. Importantly, both groups demonstrated high rates of complete gross resection (RD = 0), comparable surgical complexity, similar rates of postoperative complications, ICU admission, and length of hospital stay. The only significant difference was higher intraoperative blood loss in the DIDS group, likely reflecting chemotherapy-induced fibrosis and tissue adhesions that increase surgical difficulty. Multivariate Cox regression confirmed that residual disease was the single most important prognostic factor for both OS and PFS, with complete cytoreduction conferring major survival. The prognostic effect of residual disease was absolute: even minimal visible tumor (1–10 mm) significantly reduced OS compared with no residual disease, emphasizing the well-established principle in ovarian cancer surgery that complete gross resection (RD = 0) is of paramount importance.

Notably, when OS was stratified by FIGO stage, survival differences were not statistically significant between the two groups. This indicates that the difference in OS may be confounded by the higher proportion of stage IV patients in group B (Group A: 28.6% vs. Group B: 43.7%), even though no statistical significance was observed (*p* = 0.08321). Even though, when analyzing each stage independently, the groups become more similar in prognosis (as within-stage biology and prognosis are more homogeneous), subgroup analysis reduces the sample size for each comparison, lowering statistical power. Therefore, multivariate analysis is better to estimate the effect of the timing of cytoreductive surgery (IDS vs. DIDS). So, in multivariate analyses, FIGO stage did not independently impact survival, suggesting that in this highly selected and extensively treated cohort, complete resection outweighed disease stage.

Based on the available literature, there are only a limited number of retrospective studies that have examined the role of DIDS in advanced epithelial ovarian cancer. In 2011, Stoeckle et al. [15] conducted a retrospective study comparing early IDS (after 3–4 cycles of NACT), late IDS (after ≥5 cycles), and primary debulking surgery (PDS). They reported higher optimal resection rates in late IDS (58% vs. 36%) and a trend toward improved survival in late IDS (median 37 vs. 22 months for early IDS), though not statistically significant (*p* = 0.09). Their data suggest that extended NACT may facilitate higher rates of complete resection in select patients, and survival is not clearly compromised by the additional chemotherapy cycles. Our study also observed comparable complete resection rates between groups, even higher (>70%) in IDS group. In addition, no significant difference was found in PFS, but we did note a significantly worse OS for the DIDS group. These differences may be explained by the fact that the total number patients included in the early IDS group was only one-third compared to the late IDS (33 vs. 104 patients) group and, from the very low percentage of incomplete gross resection (RD > 0) in both groups and the fact that surgery complexity was not assessed in this study.

Similarly, in 2019, a Japanese retrospective study [16] compared IDS (after three cycles of NACT plus three cycles postoperative chemotherapy) with DIDS (after six cycles of NACT, without adjuvant chemotherapy). There were no significant differences in PFS or OS between the two groups. The strongest prognostic predictors were the ability to achieve complete resection and administration of at least six total chemotherapy cycles. The authors suggest that additional chemotherapy before debulking surgery may be an acceptable option when complete gross resection (RD = 0) is unlikely after three cycles of NACT. This matches our findings of comparable surgical and oncologic outcomes between the two groups, reinforcing that the timing of debulking is less important than achieving complete resection. However, the decreased OS was not observed in the study of Yoneoka et al. This could be due to the very low number of patients in the DIDS group (*n* = 20) and the fact that surgical complexity was also not investigated in this study.

Moreover, in 2020, two retrospective studies were published. The first one was an international, multicenter study [17] focusing exclusively on patients undergoing DIDS (after at least five NACT cycles). Plett et al. showed that when complete resection was achieved, median OS was significantly higher (49.2 months) compared to incomplete resection (33 months). Importantly, the number of NACT cycles, patient age, comorbidities, and stage of disease did not affect survival, and only complete resection was the primary determinant. The second one was a large Australian cohort [18] investigating IDS and DIDS, and found no statistically significant difference in median OS between the two groups (IDS: 41 months vs. DIDS: 43 months), provided complete cytoreduction was achieved. These results support the notion that, for patients not initially suitable for interval debulking, delayed surgery does not confer a survival penalty if complete gross resection (RD = 0) can be achieved. These nuanced messages align with the findings of our study, which found that the absence of residual disease (i.e., achievement of complete resection) was a key factor in both groups, regardless of timing. However, the difference from Yao et al., with the higher risk of death in the DIDS group, could be observed due to the unbalanced population between the groups (IDS: 87.1% vs. DIDS: 12.9%) and the lack of information about surgical complexity.

Recently, in 2023, Nasioudis et al. [19] used the U.S. National Cancer Database and compared standard (after 3–4 NACT cycles) versus delayed IDS (after 5–6 NACT cycles) for advanced high-grade epithelial ovarian cancer. Their main finding was that there was no statistically significant difference in OS between the two groups, even after adjustment for confounders, and no difference in complete resection rates. The DIDS group had less complex surgery and lower readmission rates, suggesting a clinical niche for extended NACT in frailer or less operable patients. These results generally match our findings on comparable surgical outcomes and similar PFS, but diverges regarding OS, as our findings suggest a significant OS disadvantage with DIDS. This difference could be due to the high percentage of important missing data (e.g., residual disease or recurrence), because all relevant information was extracted from a National Cancer Database. This fact can potentially lead to residual confounding and an underestimation of the effect of surgical timing on long-term survival. Thus, while overall conclusions regarding immediate surgical safety are consistent, our results more strongly support the importance of timely IDS in maximizing OS.

In contrast, a large international retrospective cohort study [20], published in 2024, evaluated the impact of receiving five or more cycles of NACT versus standard IDS (after 3–4 cycles) in advanced ovarian cancer. Gaba et al. found that DIDS (≥5 NACT cycles) was associated with significantly worse OS compared to IDS after 3–4 cycles, even when a complete resection was achieved. This study emphasizes that early, maximally effective surgery (preferably after 3–4 cycles of NACT) provides optimal survival outcomes, and that delays lead to poorer prognosis even if cytoreduction is successful. These findings are in agreement with our results that although there was no statistically significant difference in PFS between IDS and DIDS groups, a worse median OS for the DIDS group was observed. These results caution against prolonged NACT, unnecessary delay of IDS, and emphasize the need for careful multidisciplinary evaluation to optimize both timing and extent of surgery, because it should not be unduly delayed once operability is established.

The strengths of this study include its prospective systematic collection of surgical and oncological variables with no missing values and relatively long median follow-up, which allows robust survival analysis. The use of multivariate analyses provides added evidence to the ongoing debate regarding the optimal timing of interval debulking. Furthermore, this study was conducted in an ESGO-certified center for advanced ovarian cancer surgery, ensuring a high-level of quality indicators and surgical proficiency, which strengthens the credibility and impact of our study in the international literature. On the other hand, limitations include its retrospective design and risk of selection bias, as the decision to proceed with additional cycles or delay surgery may reflect unmeasured factors such as physician judgment or patient frailty. Additionally, external events such as the COVID-19 pandemic may have affected treatment pathways. The absence of randomization and the potential for variation in supportive care protocols may slightly limit the generalizability of the results.

The principal contribution of this study to the literature is to demonstrate that DIDS can achieve comparable PFS to IDS when complete resection is achieved, but OS appears compromised if surgery is delayed. Residual disease remains the overriding determinant of prognosis, highlighting the need for timely, meticulous surgery. Our findings reinforce evidence from the TRUST trial for PDS vs. IDS, that maximal cytoreduction confers survival benefit irrespective of timing, and that surgery should be pursued as early as feasible when complete resection is achievable [7]. Moreover, the fact that our findings come from an ESGO-accredited center further strengthens the results of a recent large multicenter study, with included data from various centers with no control for any quality indicator for the management of advanced ovarian cancer.

## 5. Conclusions

DIDS appears to be a safe alternative to IDS, for carefully selected patient subgroups in whom a more tailored clinical approach is warranted. Patients for whom immediate interval debulking is not feasible should undergo thorough evaluation to optimize surgical fitness and enable maximal resection at the earliest possible time. These findings highlight the importance of multidisciplinary discussion case by case and precise patient selection, balancing disease biology, surgical risk, and timing. They also address a critical gap in understanding the trade-off between delaying surgery to achieve optimal operative conditions versus the biological risks associated with prolonged chemotherapy. Future studies should aim to conduct prospective trials to further characterize and stratify patient subsets most likely to benefit from extended NACT and DIDS, particularly in light of evolving patient demographics and the expanding role of maintenance therapies.

## Figures and Tables

**Table 1:** Baseline characteristics of all cohort patients.

		Number of Patients (N)	Percentage (%)
Age (years)		mean: 60.8	SD: 12.4
BMI (kg/m^2^)		mean: 28.1	SD: 5.6
CCI		median: 2	IQR: 1–3
	0–2	73	58.4
	3–4	34	27.2
	≥5	18	14.4
NACT cycles			
	3–4	77	61.6
	≥5	48	38.4
FIGO stage			
	III	82	65.6
	IV	43	34.4
Aletti score		median: 4	IQR: 3–6
	≤3	50	40
	4–7	59	47.2
	≥8	16	12.8
Histological types			
	Serous	88	70.4
	Endometrioid	17	13.6
	Clear-cell	9	7.2
	Undifferentiated	7	5.6
	Mixed	4	3.2
CA-125 (U/mL)		median: 32	IQR: 14.7–117
Intraoperative blood loss (mL)		median: 300	IQR: 200–500
Surgery duration (min)		median: 230	IQR: 180–300
Clavien–Dindo classification		median: 24.2	IQR: 12.2–32
	Grade ≥ III	15	12
ICU admission		28	22.4
Hospital stay (days)		median: 8	IQR: 7–9
Residual disease (cm)			
	0	91	72.8
	<1	24	19.2
	≥1	10	8

**Table 2:** Comparison based on NACT cycles (IDS vs. DIDS).

**Characteristics**		**Group A (IDS)** **77 (61.6%)**	**Group B (DIDS)** **48 (38.4%)**	***p*-Value**
Age (years) mean (SD)		59.7 (13)	62.6 (11.3)	0.2036
BMI (kg/m^2^) mean (SD)		28.1 (5.6)	28.2 (5.8)	0.9302
CCI median (IQR)		2 (1–3)	2 (1–4)	0.6319
FIGO stage				0.08231
	III	55 (71.4%)	27 (56.3%)	
	IV	22 (28.6%)	21 (43.7%)	
Aletti score median (IQR)		4 (3–5.75)	4 (2–6)	0.8825
Histological types				0.8831
	Serous	55 (71.4%)	33 (68.8%)	
	Other	22 (28.6%)	15 (31.2%)	
CA-125 (U/mL) median (IQR)		34.2 (12.2–30.2)	24.9 (11.7–111)	0.2841
Intraoperative blood loss (mL) median (IQR)		300 (200–500)	450 (300–525)	**<0.05**
Surgery duration (min) median (IQR)		210 (150–300)	240 (180–330)	0.176
Clavien–Dindo classification median (IQR)		22.6 (12.2–32)	24.2 (15–32)	0.4524
Clavien–Dindo classification Grade ≥ III		9 (60%)	6 (40%)	0.754
ICU admission				0.1519
	Yes	14 (18.2%)	14 (29.2%)	
	No	63 (81.8%)	34 (70.8%)	
Hospital stay (days) median (IQR)		8 (7–9)	8 (6–10)	0.867
Residual disease (cm)				0.5791
	0	54 (70.1%)	37 (77.1%)	
	<1	17 (22.1%)	7 (14.6%)	
	≥1	6 (7.8%)	4 (8.3%)	

**Table 3:** Cox regression for recurrence for all cohort patients.

		Univariate			Multivariate		
		HR	95% CI	*p*-Value	HR	95% CI	*p*-Value
Age (years)		1.01	0.99, 1.02	0.587			
BMI (kg/m^2^)		1.01	0.96, 1.07	0.688			
CCI		1.08	0.97, 1.29	0.196	1.09	0.98, 1.22	0.104
NACT cycles							
	3–4	0.88	0.57, 1.36	0.56			
	≥5	1	1	1			
FIGO stage							
	III	1	1	1	1	1	1
	IV	1.69	1.07, 2.68	**0.0258**	1.48	0.90, 2.42	0.12
Aletti score		1.14	1.02, 1.27	**0.0167**			
Histological types							
	Serous	0.90	0.40, 2.02	0.796			
	Other	1	1	1			
CA-125 (U/mL)		1	1, 1	0.051			
Intraoperative blood loss (mL)		1	0.99, 1.001	0.419			
Surgery duration (min)		1.004	1.001, 1.01	**<0.01**			
Clavien–Dindo classification		1.02	1.007, 1.04	**<0.01**	1.03	1.01, 1.05	**<0.01**
ICU							
	Yes	1.32	0.77, 2.25	0.317			
	No	1	1	1			
Hospital stay (days)		1.03	0.98, 1.10	0.263			
Residual disease (cm)							
	0	1	1	1	1	1	1
	<1	1.18	0.71, 1.95	0.532	0.97	0.57, 1.66	0.920
	≥1	3.51	1.68, 7.35	**<0.01**	2.84	1.33, 6.06	**<0.01**

**Table 4:** Cox regression for death for all cohort patients.

		Univariate			Multivariate		
		HR	95% CI	*p*-Value	HR	95% CI	*p*-Value
Age (years)		1.02	0.99, 1.07	0.094	1.02	0.99, 1.04	0.058
BMI (kg/m^2^)		0.99	0.94, 1.05	0.78			
CCI		0.97	0.87, 1.09	0.614	1.11	0.96, 1.28	0.160
NACT cycles							
	3–4	1	1	1	1	1	1
	≥5	1.83	1.16, 2.9	**<0.01**	1.84	1.15, 2.96	**0.0116**
FIGO stage							
	III	1	1	1			
	IV	1.45	0.90, 2.34	0.13			
Aletti score		1.04	0.93, 1.16	0.477			
Histological types							
	Serous	0.75	0.22, 2.49	0.633			
	Others	1	1	1			
CA-125 (U/mL)		1	1, 1.001	**0.018**			
Intraoperative blood loss (mL)		1.001	1, 1.001	**<0.01**			
Surgery duration (min)		0.99	0.99, 1.002	0.63			
Clavien–Dindo classification		1.01	0.99, 1.03	0.181	1.02	1.002, 1.04	**0.0472**
ICU							
	Yes	1.13	0.64, 2.01	0.674			
	No	1	1	1			
Hospital stay (days)		0.96	0.90, 1.03	0.247			
Residual disease (cm)							
	0	0.23	0.11, 0.47	**<0.01**	0.18	0.08, 0.38	**<0.01**
	<1	0.53	0.24, 1.19	0.125	0.48	0.21, 1.11	0.085
	≥1	1	1	1	1	1	1

## Data Availability

The data presented in this study are available on request from the corresponding author, because they are not publicly available due to privacy or ethical restrictions.

## Abbreviations

The following abbreviations are used in this manuscript:

DIDS	Delayed interval debulking surgery
IDS	Interval debulking surgery
NACT	Neoadjuvant chemotherapy
BMI	Body mass index
ICU	Intensive care unit
FIGO	International Federation of Gynecology and Obstetrics
PDS	Primary debulking surgery
RECIST	Response Evaluation Criteria in Solid Tumors
KELIM	CA-125 elimination rate constant K
CCI	Charlson Comorbidity Index
RD	Residual disease
OS	Overall survival
PFS	Progression-free survival
COVID-19	Coronavirus Disease 2019
